# Prevalence of postpartum depression and associated factors among postnatal care attendees in Debre Berhan, Ethiopia, 2018

**DOI:** 10.1186/s12884-020-02873-4

**Published:** 2020-03-30

**Authors:** Abate Dargie Wubetu, Nigus Alemnew Engidaw, Kefyalew Dagne Gizachew

**Affiliations:** Department of Psychiatry, College of Health Science and Medicine, Debre Berhan University, P.O. Box 445, Debre Berhan, Ethiopia

**Keywords:** Prevalence, Magnitude, Puerperium, Depression, Morbidity, Ethiopia

## Abstract

**Background:**

Postpartum depression explains various groups of depressive symptoms and syndromes that can take place during the first 6 weeks following birth. The postpartum period is a critical time where both mild and severe mood disorders can occur. The familiar forms are baby blues and postpartum depression. Understanding the prevalence and associated factors of postpartum depression is mandatory for early detection and treatment.

**Methods:**

Institution based cross-sectional study was conducted from 1st May to June 30, 2018. The study participants were eligible women who came to Debre Berhan referral hospital and health centers for postnatal care and vaccination service. The Edinburgh postnatal depression scale was used to assess postpartum depression. A systematic random sampling technique was used to collect the data after determining the skip fraction (k = 2). The collected data were coded and entered into Epi-info version 7 and transported to SPSS version 20 for analysis. Both bivariate and multivariate binary logistic regression were done to identify associated factors. During bivariate analysis, variables with *p*-value < 0.05 were included in multivariate analysis. Odds ratios and their 95% confidence intervals were computed and variables with p-value less than 0.05 were considered to declare significantly associated factors (multivariate analysis).

**Results:**

A total of 308 mothers who attended postpartum care we're included, which was a 100% response rate. The prevalence of postpartum depression was found to be 15.6% (95%CI = 11.7, 19.8). Being widowed/widower, having poor social support, having a current hospitalized child, and experienced a death of family member or close relative were significantly associated with postpartum depression.

**Conclusions:**

The prevalence of postpartum depression was lower than most studies done in different areas. Major life events and traumas are associated with an increased risk of postpartum depression. Health professionals should be aware of the mother’s circumstances during the puerperium, they should initiate support to reduce the risk of depression in the postpartum period. Health care professionals working postpartum care clinics should give special attention to mothers who are widowed/widower, have poor social support, have a current hospitalized children, and experienced a death of family member or close relative.

## Background

Postpartum Depression (PPD) refers to non-psychotic depressive episodes that begin in or extend into the postpartum period [1]. According to The American Psychiatric Association (APA) postpartum depression is defined as the occurrence of a Major Depressive Episode (MDE) within 4 weeks after delivery [2].

About 14% of the worldwide burden of disease has been attributed to neuropsychiatric disorders, including those disorders that can occur during the postpartum period. Such estimates have drawn attention to the importance of mental disorders for public health [3]. The estimated lifetime prevalence of having one or more of the mental disorders varies widely across the world as shown by mental health surveys, from 12.1% in Nigeria to 47.4% in the United [4].

Postpartum depression is a non-psychotic depressive disorder that affects 13 to 19% of postpartum women and those women experience signs and symptoms like self-blaming thought, guilt about their inability to look after their new baby, low self-esteem, lack of interest in one’s environment, insecurity and suicidal thoughts. This condition begins in the postpartum period and persists up to a one-year duration after delivery. The treatment option for PPD women is behavioral counseling and anti-depressant therapy [2, 5–7].

World Health Organization (WHO) reported that for women of reproductive age group depression becomes the leading cause of disease burden [8]. Postpartum nonpsychotic depression is a considerable public health problem and the most common complication of childbearing age that affect approximately 10–15% of postpartum women. In developing countries, the prevalence of postpartum depression almost doubled that of the developed world. The effect of postpartum depression on the mother, her marital relationship and her children make it an important condition to diagnose, treat and prevent [9, 10]. Untreated postpartum depression can have a prolonged adverse effect on the mother and her children. Pregnant mothers’ ongoing depression can contribute to emotional, behavioral, cognitive and interpersonal problems [11].

Epidemiological studies conducted in China, Japan, India and New Dubai Hospital in Dubai, revealed that the overall prevalence of postpartum depression was 13.5, 17, 23, and 15.8% respectively [12–15]. Another quasi-experimental study conducted among 420 consenting pregnant women on the title of postpartum depression in peri-urban communities of Karachi, Pakistan, revealed that the overall prevalence of postpartum depression was 28.8% [16].The growth of the child is potentially affected in response to the potential decline in care by the mother experienced PPD. Determining the prevalence of postpartum depression, and identifying associated factors with it is important to show the magnitude of the problem. This study aimed to determine the prevalence of postpartum depression in the study area and to identify associated factors of postpartum depression.

### Specific objectives

- To determine the prevalence of postpartum depression in postnatal care attendees
- To identify factors associated with postpartum depression in postnatal care attendees.

## Methods

### The study area, design and period

The study was conducted in Debre Berhan town which found in the North Shoa zone at Amhara regional state of Ethiopia. This town is found 130 km away from the capital city of Ethiopia; Addis Ababa. The cross-sectional study design was employed from 1st May to June 30, 2018. The study site had a total of one government-owned referral hospital, three health centers, five private clinics, and more than ten pharmacies. There were 613 mothers who gave birth and attended postpartum care and vaccination service during the study period.

### Population

Source Population: All women who came for postnatal care and vaccination services within 6 weeks after delivery in a referral hospital and health centers in Debre Berhan, Town Ethiopia.

Study Population: All women who came for postnatal care and vaccination service within 6 weeks after delivery during the data collection period.

### Eligibility

#### Inclusion Criteria

All women who gave birth and who came for postnatal care and vaccination service within 6 weeks after delivery in health centers and referral hospital were included.

#### Exclusion Criteria

Women who had a verbal communication problem and complete loss of hearing were excluded.

### Sample size calculation and sampling technique

The required sample size was determined by using a single population proportion formula with the following assumptions: (Z α/2) = value for the 95% CI, =1.96, the proportion of postpartum depression; similar study at Gondar, Ethiopia (*P* = 23%) [17], d = margin of error taken as 5%; by adding 10% of study subjects as nonresponse rate, the final sample size became 308. The study subjects were interviewed by using systematic random sampling after determining the sampling fraction (k = 613/308 = 2) and the first participant was selected by using the lottery method. The total sample size (*n* = 308) was allocated proportionally according to the total number of postpartum care and vaccination service attendees at each health center (district 04, district 07, district 08) and Debre Berhan referral hospital.

### Study variables

#### Dependent variable

Postpartum Depression (yes/no).

#### Independent variables

Socio-demographic factors: (age, educational status, economic, marital status, employment, monthly income, current residence).

Social factors: - social and husband support, emotional violence, physical violence, sexual violence.

Substance use: use of any substance during the puerperium period for a non-medical purpose (like Khat, alcohol, and cigarette).

Obstetrics factors: parity, pregnancy intention, currently a hospitalized child, mode of delivery, perinatal complication or illness, stressful life event during the puerperium period and undesired fetal sex.

Previous psychiatric history: A family history (first-degree relatives) of psychiatric problems.

### Operational definitions

#### Poor social support

Mothers who scored 3–8 on the (Oslo-3) social support scale during puerperium.

#### Moderate social support

Mothers who scored 9–11 on the (Oslo-3) social support scale during puerperium.

#### Strong social support

Mothers who scored 11–14 on the (Oslo-3) social support scale during puerperium.

### Data collection tools and procedures

A structured interviewer-administered questionnaire was used to collecting information from study participants. Sociodemographic, clinical, and obstetric factors were assessed by predefined checklists. The social support level was assessed by using the Oslo social support scale, and the Edinburgh Postnatal Depression Scale (EPDS) was used to assess postpartum depression. Data were collected with an interviewer-administered questionnaire from mothers who came for postnatal care and vaccination service.

### Data quality control and analysis

The data collection instrument was pre-tested on 5% of the sample size out of Debre Berhan town to improve language clarity and appropriateness of data collection tools. The estimated time required, and necessary amendments were made after the piloting of the questionnaire. Four fourth-year undergraduate nursing students collected the data. The data collectors were trained for 1 day on the techniques of data collection. The training also included the importance of disclosing the possible benefit and purpose of the study to the study participants before the start of data collection. The researcher checked completeness and consistency of questionnaires filled by the data collectors to ensure the quality of data and also visited the data collectors as many times as possible to check whether he/she collected the data appropriately. The collected data were entered into Epi-info version 7 and analysis was done after the data were imported to SPSS version 20. During bivariate analysis, variables with *p*-value < 0.05 were exported to multivariate analysis. Crude and adjusted odds ratios were analyzed using bivariate and multivariable binary logistic regression analysis and the level of significance of association was determined at p-value < 0.05.

## Results

### Socio-demographic characteristics of postpartum mothers

There were 613 mothers who gave birth and attended postpartum care and vaccination service during the study period. Among them, 308 mothers were included in the study by using systematic random sampling technique, which was a 100% response rate. Among the study subjects, 286 (86%) were aged 25–45 years and almost 85% were married. The majority of the participants, 206(66.9%) had attended formal (modern) education. Regarding ethnicity, the majority of the study participants, 234(76%) were Amhara and 62(20.1%) were Oromo. Two hundred sixty-eight (87%) of the participants earn a monthly income greater than 2500 Ethiopian Birr. Almost 60 % of the participant’s religion, 191(62%) were orthodox Christian followers (Table 1).

**Table 1:** Socio-demographic characteristics of mothers who have postnatal care at Debre Berhan health centers and referral hospital, 2018

Variables	Category	Frequency	Percentage
Age in years	19–24	43	14.0
	25–45	265	86.0
Marital status	Single	28	9.1
	Widowed/widower	19	6.2
	Married	261	84.7
Address	Urban	215	69.8
	Rural	93	30.2
Religion	Orthodox	191	62.0
	Catholic	15	4.9
	Muslim	47	15.3
	Protestant	55	17.9
Ethnicity	Amhara	234	76.0
	Oromo	62	20.1
	Tigray	12	3.9
Attended modern education	Yes	206	66.9
	No	102	33.1
Occupational status of mother	Government employed	84	27.2
	House wife	146	47.4
	private employed	58	18.8
	Farmer	8	2.6
	Unemployed	12	3.9
Monthly income	≤ 1200	5	1.6
	1201–2500	35	11.4
	≥2501	268	87.0

### Obstetric and clinical characteristic of postpartum mothers

From 308 study participants, the majority of respondents 254(82.5%) were multigravida (give birth > 1) and 54(17.5%) were primigravida (having a first child). Almost 80% of participants had two or more living children during the study period. Regarding termination of pregnancy, 53(17.2%) had experienced termination and 39(12.7%) had experienced the death of their child. Forty-eight (15.6%) participants reported that the recent pregnancy was unplanned. Moreover, the sex of the last baby 189(61.4%) were male and the rest were female. Regarding the desired sex of the last baby, 36(11.7%) of the respondents said that the sex of their infant was unwanted gender. Nearly 62% of participants, 190(61.7%) mode of delivery was a spontaneous vaginal delivery. Forty-seven, 47(15.3%) respondents had suffered from any diagnosed illness during their last pregnancy and 95(30.8%) study mothers reported their babies were admitted to the hospital at least once before (Table 2).

**Table 2:** Obstetric and clinical characteristic among mothers who have postnatal care, Debre Berhan, Ethiopia, 2018

Variable	Category	Frequency	Percent %
Number of pregnancy	1	54	17.5
	≥ 2	254	82.5
Living child	1	64	20.8
	≥ 2	244	79.2
Sex of last baby	Male	189	61.4
	Female	119	38.6
Desired sex of the baby	Desired	271	71.3
	Undesired	109	28.7
Abortion	Yes	25	6.6
	No	355	93.4
Pattern of pregnancy termination	Spontaneous	47	15.3
	Induced	6	1.9
Number of termination of pregnancy	1	49	15.9
	≥ 2	4	1.3
Baby death	Yes	38	10.0
	No	342	90.0
Had a current hospitalized baby	Yes	95	30.8
	No	213	69.2
Mode of delivery	Vaginal	190	61.7
	Cesarean section	87	28.2
	Vacuum/forceps	31	10.1
Planed pregnancy	Yes	260	84.4
	No	48	15.6
Illness/complication in last pregnancy	Yes	47	12.4
	No	333	87.6

### Psychosocial factors (in last 6 months) of postpartum mothers

From the total study participants, 62(20.1%) responded that their family or a close relative had died. Almost one fifth (19.5%), participants reported that there was a serious illness, injury or assault during the recent pregnancy. Almost 60, 59(19.2%) study participants had experienced parent or child death and 42(13.6%) participants reported that they were separated due to marital difficulty. In addition, 41(13.7%) study participants were unemployed / not been able to work in the last 6 months of the study period. Moreover, 40(13%) reported physical violence during the last pregnancy (Table 3).

**Table 3:** Psychosocial characteristic (in last 6 months) among mothers who have postnatal care, Debre Berhan, Ethiopia, 2018

Variable	Category	Frequency	Percent (%)
Serious illness injury or assault during pregnancy	Yes	42	13.6
	No	266	86.4
Close relative serious illness, injury or assault	Yes	60	19.5
	No	248	80.5
Died spouse, parent or child	Yes	59	19.2
	No	249	80.8
Died Family or close relative	Yes	62	20.1
	No	246	79.9
Major financial crisis	Yes	37	12.0
	No	271	88.0
Sacked from job	Yes	12	3.9
	No	296	96.1
Unemployed/not able to work	Yes	41	13.3
	No	267	86.7
Separation due to marital difficulty	Yes	42	13.6
	No	266	86.4
Broken off a steady relationship	Yes	33	10.7
	No	275	89.3
Serious problem with close friend, neighbor /relative	Yes	37	12
	No	271	88
Lost / stolen property which mattered a lot	Yes	30	9.7
	No	278	90.3
Any problems with police/court	Yes	18	5.8
	No	290	94.2
Emotional violence	Yes	26	8.4
	No	282	91.6
Physical violence	Yes	40	13
	No	268	87
Who physically violate you	Boy friend	21	52.5
	Family member	8	20
	Stranger	11	27.5
Forced sexual activity	Yes	35	11.4
	No	273	88.6
Who forced you for sexual activity	Boy friend	15	37.5
	Family member	2	5
	Stranger	18	45

### Substance use among postpartum mothers

Overall, 31(10.1%) of study participants reported the use of any substance before pregnancy and of these the majority of use was alcohol-related; i.e. 21(67.7%). The remaining used only Khat at least once in a lifetime. Regarding substance used during the last pregnancy, 18(5.8%) respondents used any kind of substance, and all of them used alcohol.

### History of known illness among postpartum mothers

Of the total study participants, 31(10.1%) had a known history of mental illness. In addition, 44(14.3%) study respondents had a family history of known mental illness and 28(9.1%) had diagnosed diabetes mellitus and hypertension.

### Social support among postpartum mothers

Social support status was assessed by using the Oslo-3 social support scale. From the total study participants, the majority 137(44.5%) had moderate social support, 114(37%) had poor social support and the rest had strong social support. During pregnancy, 175(56.8%), 111(36%), and 22(7.1%) had strong, moderate, and poor husband support respectively. Thirty-six percent, 112(36.4%) study participants had no practical support from a family member during pregnancy (such as cooking, washing, cleaning or child-rearing), and during puerperium.

### Prevalence of postpartum depression and its associated factors

According to the Edinburgh Postnatal Depression Scale (EDPS), study participants who scored ≥13 are considered as having postpartum depression. Hence, the prevalence of postpartum depression among mothers who have postnatal care follow up was 15.6% [95% CI = 11.7, 19.8].

Binary logistic regression was performed to assess the association of each independent variable with the outcome variable (postpartum depression). The variables that showed a significance level (*p* < 0.05) during bivariate analysis were added to the multivariate regression model. Twenty-two independent variables were shown to be significantly associated during the bivariate analysis. The result of the multivariate analysis showed that only four variables were statistically significant. Being widowed/widower, having a current hospitalized child, having died family or close relative, having poor social support showed a significant association with postpartum depression.

The results showed that women who were widowed/widower had an association with postpartum depression; and were four times more likely to experience postpartum depression than those who were married [AOR = 4.17, 95% CI = 1.14, 15.20]. In addition, respondents who had poor social support were five times more likely to be depressed than those who had strong social support [AOR = 5.11, 95% CI = 1.00, 26.18]. Respondents who had a current hospitalized children were nearly 3 times more likely to be depressed as compared to respondents who does not have a current hospitalized child [AOR = 3.32, 95%CI = 1.39,7.93]. In a similar dimension, participants who had experienced a death of a family member or close relative in the last 6 months were three times more likely to be depressed than those who did not experience this [AOR = 2.92, 95%CI = 1.01,8.50], (Table 4).

**Table 4:** Bivariate and multivariate analysis of factors associated postpartum depression among mothers who have postnatal care, Debre Berhan, Ethiopia, 2018

Variables		Postpartum Depression		COR (95%CI)	AOR(95%CI)
		Yes	No		
Marital status	Single	10	18	4.28 (1.81,10.13)	2.70 (0.72,10.21)
	Widowed/widower	8	11	5.60 (2.09,15.03)	4.17 (1.14,15.20)*
	Married	30	231	1.00	1.00
Attend modern school	Yes	24	182	1.00	1.00
	No	24	78	2.33 (1.25,4.36)	0.76 (0.34,1.71
Social support	Poor	34	80	11.69 (2.70,50.67)	5.11 (1.00,26.18)*
	Moderate	12	125	2.64 (0.57,12.19)	1.93 (0.36,10.36)
	Strong	2	55	1.00	1.00
Husband support	Poor	8	14	4.43 (1.65,11.8)	0.70 (0.14,3.57)
	Moderate	20	91	1.70 (0.870,3.33)	1.02 (0.42,2.47
	Strong	20	155	1.00	1.00
Serious illness, injury or assault to a close relative	Yes	17	43	2.77 (1.41,5.44)	0.28 (0.08,10.96)
	No	31	217	1.00	1.00
Died spouse, parent or child	Yes	20	39	4.05 (2.08,7.89	0.56 (0.17,1.85
	No	28	221	1.00	1.00
Died close family or relative	Yes	21	41	4.15 (2.15,8.04)	2.92 (1.01,8.50)*
	No	27	219	1.00	1.00
Has major financial crisis	Yes	14	23	4.24 (1.99,9.03)	1.74 (0.55,5.49
	No	34	237	1.00	1.00
Sacked from job	Yes	5	7	4.20 (1.28,13.85	0.67 (0.21,2.11)
	No	43	253	1.00	1.00
Unemployed/not able to work	Yes	15	26	4.09 (1.97,8.51)	3.21 (0.68,15.12)
	No	33	234	1.00	1.00
Separation due to marital difficulty	Yes	13	29	2.96 (1.40,6.23)	0.72 (0.22,2.40)
	No	35	231	1.00	1.00
Broken off steady friendship/relationship	Yes	14	19	5.22 (2.40,11.37)	0.75 (0.22,2.50)
	No	34	241	1.00	1.00
Serious problem with close friend, neighbor /relative	Yes	13	24	3.65 (1.70,7.83)	1.86 (0.51,6.81)
	No	35	236	1.00	1.00
Any problems with police/court	Yes	6	12	2.95 (1.05,8.30)	0.62 (0.19,2.05)
	No	42	248	1.00	1.00
Emotional/physical abused by parents	Yes	9	17	3.30 (1.37,7.92)	1.51 (0.47,4.90)
	No	39	243	1.00	1.00
Had a current hospitalized baby	Yes	27	68	3.63 (1.926,6.84)	3.32 (1.39,7.93)*
	No	21	192	1.00	1.00
Pregnancy intention	Planned	35	225	1.00	1.00
	Unplanned	13	35	2.39 (1.15,4.95)	0.75 (0.25,2.21)
Forced sexual activity	Suffered	13	22	4.02 (1.86,8.69)	2.00 (0.68,5.93)
	Not suffered	35	238	1.00	1.00
Diagnosed mental illness in the family	Yes	15	29	3.62 (1.76,7.46	2.04 (0.68,6.14)
	No	33	231	1.00	1.00
Diabetes mellitus	Yes	12	16	5.08 (2.23,11.6)	2.40 (0.69,8.35)
	No	36	244	1.00	1.00

Key: * = *p*-value less than 0.05; *COR* crude odds ratio, *AOR* adjusted odds ratio, *CI* confidence interval

## Discussion

### Prevalence of postpartum depression

There were 613 mothers who gave birth and attended postpartum care and vaccination service during the study period. Among them, 308 mothers were included in the study by using systematic random sampling technique (k = 2), which was a 100% response rate. The overall prevalence of postpartum depression was 15.6%(95%CI = 11.7, 19.8).

This was almost similar to other studies that were conducted in Delhi and adjacent states of northern India, 15.8% [18], Egypt, 17.9% [19], and Uganda, 16.3% [20].

The prevalence rates were higher in our study when compared with Canadian, Denmark, and Uganda (Kampala), and Egypt study which was 1.6, 5.5 and 6.1%, 7.14% respectively [21–24]. The higher rate might be due to the use of different measurement tools, assessment period, social support level and economic status of the mothers.

On the other hand, this figure was lower when compared with other similar studies done in Lebanon, 21% [25], Cameroon, 23.4% [26], Nigeria, 23% [27]. The lower prevalence rate in our study might be due to difference in residency, and sample size difference. For instance, the study in Lebanon was conducted in a rural area by using a follow-up study with a sample size of 396 mothers. In addition, the study conducted in Cameroon used a case-control study design while our study used a cross-sectional study design. Similar studies in Ethiopia revealed that 22.1% [28], 22.4% [29], 31.5% [30], of mothers were depressed during puerperium. These studies had higher prevalence rates than our study. The higher prevalence report in these studies might be due to the screening tool, study design, and sample size. The study done in the Oromia region used a self-reporting questionnaire (SRQ) and a community-based cross-sectional study.

### Factors associated with postpartum depression

Among the sociodemographic factors, study subjects who were widowed/widower had an association with postpartum depression: almost four times higher when compared with those who were married. In this cohort, the association was in agreement with the study done in Ethiopia [31]. The agreement might be due to the fact that being married is important for mental health; especially during the postpartum period.

In the social support dimension, respondents who had poor social support were more likely to be depressed than those who had strong social support. The association in estimation was in line with studies done in Malaysian and Pakistan [32], Cameroon; Yaoundé [26] and Hiwot Fana specialized University Hospital in Ethiopia [10]. In fact, having poor social support is one of the highest contributors to poor mental health [33].

The variables that were found to have an association with postpartum depression were having a hospitalized child during the postpartum period. Respondents who had a current hospitalized child were almost three times more likely to be depressed as compared to the respondent who had not a current hospitalized child. In a similar dimension, participants who had experienced a death of a family member or close relative in the last 6 months were three times more likely to have postpartum depression than those who does not experienced a death of a family member or close relative. The association was in agreement with the study done in Robe town; Bale Zone, Ethiopia [30]. The possible reason might be due to the fact that experiencing life-threatening events during the postpartum period became intolerable and may affect the mental wellness of the mothers.

### Limitations

Postpartum women with persisting depression already acquired before/during pregnancy were not excluded and this may further increase the prevalence rates of postpartum depression. The study only included mothers who had postnatal care follow up in the urban area. Since we recruited multiple data collectors, there may be interviewer bias.

## Conclusions

Though significant proportions of postnatal mothers had depression, the prevalence of postpartum depression was lower than most studies in different areas. Major life events and trauma are associated with an increased risk of postpartum depression. Health professionals should be aware of the mother’s circumstances during pregnancy. They should initiate support to reduce the risk of depression in the postpartum period. Health care professionals working in maternal and child health clinics should give special attention to pregnant mothers who are widowed/widower, have poor social support, have a current hospitalized child, and experienced a death of a family member or close relative.

### Recommendations

It would be advisable if midwife professionals routinely screen postpartum depressive symptoms and link them to mental health services just like other reproductive health problems for mothers attending hospitals and health centers after delivery.

## Data Availability

The datasets used and/or analyzed during the current study are available from the corresponding author on reasonable request.
